# Clinicopathological features and prognosis of patients with de novo versus nevus-associated melanoma in Taiwan

**DOI:** 10.1371/journal.pone.0177126

**Published:** 2017-05-04

**Authors:** Yi-Shuan Sheen, Yi-Hua Liao, Ming-Hsien Lin, Jau-Shiuh Chen, Jau-Yu Liau, Cher-Wei Liang, Yih-Leong Chang, Chia-Yu Chu

**Affiliations:** 1 Graduate Institute of Pathology, National Taiwan University College of Medicine, Taipei, Taiwan; 2 Department of Dermatology, National Taiwan University Hospital and National Taiwan University College of Medicine, Taipei, Taiwan; 3 Graduate Institute of Clinical Medicine, National Taiwan University College of Medicine, Taipei, Taiwan; 4 Department of Surgery, National Taiwan University Hospital Hsin-Chu Branch, Hsin-chu, Taiwan; 5 Department of Pathology, National Taiwan University Hospital, Taipei, Taiwan; University of Queensland Diamantina Institute, AUSTRALIA

## Abstract

Studies surveying melanomas associated with melanocytic nevi in Asia are rare. In this study, we examined whether nevus-associated melanomas differ from de novo melanomas in terms of their associations with clinical factors, histologic characteristics, and patient survival in Taiwan. Using data on cancer cases obtained from the Department of Pathology archives and the Cancer Registry of National Taiwan University Hospital, we conducted a retrospective analysis of 103 consecutive melanoma patients who were diagnosed between 2010 and 2015 and received follow-up through November 2016. Approximately 17.5% of the melanomas in question were associated with a nevus. In patients under 65 years of age, non-acral lentiginous melanomas were significantly associated with a higher percentage of nevus-associated melanomas. The superficial spreading subtype, younger patient age, thinner tumor, intermittent solar exposure, and early stage were significant predictors of a melanoma being histologically associated with a nevus. The appearance of a nevus associated with a melanoma predicted better recurrence-free survival compared with de novo melanomas. Although acral lentiginous melanomas (70.9%) constituted the most common histologic subtype, only 9.6% of the acral lentiginous melanomas were associated with a nevus. Furthermore, there was no statistically significant difference between the nevus-associated and de novo acral lentiginous melanomas with regard to clinicopathological factors and survival. In conclusion, nevus-associated melanomas were uncommon among acral lentiginous melanomas. Relatedly, because over half of all melanomas in Asians are acral lentiginous melanomas, Asians are less likely than Caucasians to have nevus-associated melanomas.

## Introduction

According to Ackerman’s large series of malignant melanomas in Caucasians, approximately 80% of the cases arose de novo, while only about 20% of the cases, most of which were located on the trunk and proximal extremities, developed in association with pre-existing melanocytic nevi [1, 2]. Although all melanoma subtypes may be associated with nevi, lentigo malignant melanomas (LMMs) and acral lentiginous melanomas (ALMs) are less likely to arise from nevi than are superficial spreading melanomas (SSMs) and nodular melanomas (NMs) [2]. In Asia, more than fifty percent of melanomas have been reported to be ALMs, while melanomas on sun-exposed areas appeared less frequently in Asians than in Caucasians [2, 3]. Relatedly, ultraviolet radiation does not appear to be associated with melanomas in black and Asian populations [4].

Statistical data regarding melanomas associated with melanocytic nevi in Asia appears to be limited, although we did discover such information with regard to populations in the West [2]. To our knowledge, no studies of Asian populations have included a comparison of de novo and nevus-associated melanomas during a given time period [2]. Meanwhile, ALMs, which are rarely associated with melanocytic nevi, account for more than half of all melanomas in Asians [2]. Therefore, we conducted this study in order to determine whether any differences exist between Asians and Caucasians in the frequency of de novo and nevus-associated melanomas and to determine the proportion of nevus-associated ALMs at our own hospital. In addition, the associations between nevi and overall survival (OS), distant metastasis-free survival (DMFS), and recurrence-free survival (RFS), as well as various factors that have been found to affect survival, have rarely been discussed in the existing literature [5–8]. In this study, therefore, we correlated the presence of nevi with clinicopathological variables and prognosis in order to further explore the associations of melanocytic nevi in a large cohort of melanoma patients in Asia.

## Materials and methods

### Patients and tissues

This retrospective cohort study was authorized by the Research Ethics Committee of National Taiwan University Hospital (NTUH-REC No.: 201603007RIND) and was conducted according to the principles of the Declaration of Helsinki. One hundred and sixty-four consecutive pathology reports regarding cases of melanomas in situ and invasive cutaneous melanomas diagnosed between January 1, 2010, and December 31, 2015, at the National Taiwan University Hospital Department of Pathology were obtained using a computer-assisted search. All the cases were reviewed by at least 2 pathology faculty members at the time of diagnosis. A standard protocol, through which the reviewing pathologist comments on melanoma subtypes, mitotic count, ulceration, lymphovascular invasion, desmoplastic components, neurotropism, predominant cell type, regression, tumor infiltrating lymphocytes, Breslow thickness, and the presence or absence of an associated nevus, is followed in the hospital’s Department of Pathology. Pathology reports from the study period were evaluated, while biopsies from specimens that did not allow the lesion to be assessed precisely were omitted (n = 23). Melanomas representing a local recurrence or metastasis (n = 21) were also excluded from the analysis, as well cases lacking adequate medical history data (n = 17). After excluding the abovementioned cases, pathology reports for 103 melanomas remained. All patients who enrolled in the study gave written informed consent to use their resected tissues and received standard-of-care therapy [9]. For each melanoma, data were collected on the histologic subtype, Breslow thickness, mitotic count, ulceration, lymphovascular invasion, desmoplastic component, neurotropism, cell type, regression, and tumor infiltrating lymphocytes, as well as whether a nevus was associated with the melanoma, and, if so, the nevus type according to the pathology reports [5, 10]. A nevus-associated melanoma was diagnosed based on the presence of histopathological evidence of both a melanocytic nevus and a melanoma in surgically obtained specimens. A de novo melanoma was defined as a melanoma not accompanied by histopathological evidence of a preexisting nevus. The histologic melanoma subtypes were classified as ALMs, SSMs, NMs, LMMs, mucosal melanomas, and desmoplastic melanomas. According to a recent study [11], the mitotic count was categorized into groups of <1 mitosis/mm^2^, 1–5 mitoses/mm^2^, and ≥ 6 mitoses/mm^2^. Lesions distributed on the trunk and extremities were acknowledged to be distributed on areas receiving intermittent sun exposure; lesions on the head and neck were regarded as being located on areas receiving chronic sun exposure; and the acral and genital areas were recognized as non-sun-exposed areas [12]. For each patient, clinical data describing the patient demographics, the American Joint Committee on Cancer stage, the clinical course, and follow-up through November 30, 2016, were assessed from the records and other data provided by the Cancer Registry of the Medical Information Management Office of National Taiwan University Hospital. OS was defined as the time from the diagnosis until the last follow-up visit or death [5]. RFS was defined as the length of time after treatment during which no local recurrence or regional or distant metastasis was detected. Finally, DMFS was defined as the length of time after therapy during which no distant metastasis was noted.

### Statistical analysis

The raw data were summarized by descriptive statistics. Associations between nevus-associated melanomas and clinicopathological factors were analyzed with Fisher's exact test or the chi-square test. Specifically, Fisher’s exact test was used instead of the chi-square test for expected cell counts of less than 5. Survival probabilities were assessed using the Kaplan-Meier method and then evaluated by log-rank tests [9]. The influence of each factor on survival was calculated using univariate analysis and the Cox proportional hazards regression models. However, given that mitotic count, thickness, ulceration, and lymph node metastasis are aspects of a cancer’s stage, the stage was not considered in the Cox regression models. All statistical tests were two-sided, and a *p*-value of 0.05 or less was regarded to be statistically significant. All the statistical analyses were performed by an independent statistical analyst using SAS 9.4 (Cary, North Carolina, USA).

## Results

Tissues from a total of 103 cases with primary melanoma were investigated (S1 Table). These patients consisted of 53 men and 50 women with a mean age of 65 years (median: 68 years; range: 21–88 years) (Table 1). Most of the melanomas (73/103, 70.9%) were located on the palms and soles. Ulceration was present in 29.1% (30/103) of the cases. The mean and median Breslow thicknesses of the melanomas were 3.7 and 2.1 mm (range 0.25–42 mm). At the time of presentation, 11.7% of the subjects had a melanoma in situ, 71.8% were at stage I or stage II, and 16.5% were at stage III or stage IV. The mean and median durations of follow-up after the diagnosis of melanoma were 2.6 and 2.5 years, respectively.

**Table 1:** Clinical and histologic characteristics.

Characteristic	Total	De novo	Nevus-associated	*P*-value
Age (years)				
<65	45	33 (73.3%)	12 (26.7%)	0.03^a^*
≥65	58	52 (89.7%)	6 (10.3%)	
Sex				
Female	49	39 (79.6%)	10 (20.4%)	0.46^a^
Male	54	46 (85.2%)	8 (14.8%)	
Site				
Trunk	11	6 (54.5%)	5 (45.5%)	
Head/neck	2	2 (100%)	0 (0.0%)	
Extremities	12	6 (50%)	6 (50%)	0.001^b^*
Acral	73	66 (90.4%)	7 (9.6%)	
Special site	5	5 (100%)	0 (0.0%)	
Solar exposure				
No	78	71 (91%)	7 (9%)	
Intermittent	23	12 (52.2%)	11 (47.8%)	<0.001^b^*
Chronic	2	2 (100%)	0 (0.0%)	
Subtypes				
SSM	21	12 (57.1%)	9 (42.9%)	
NM	6	4 (66.7%)	2 (33.3%)	
Acral	73	66 (90.4%)	7 (9.6%)	0.004^b^*
Mucosal	2	2 (100%)	0 (0.0%)	
Desmoplastic	1	1 (100%)	0 (0.0%)	
Thickness of invasive melanomas (n = 91), mm				
≤ 1.0	23	15 (65.2%)	8 (34.8%)	0.06^b^
1.01–2.0	22	19 (86.4%)	3 (13.6%)	
2.01–4.0	24	19 (79.2%)	5 (20.8%)	
>4.0	22	21 (95.5%)	1 (4.5%)	
Mitosis/mm^2^				
<1	30	23 (76.7%)	7 (23.3%)	
1–5	57	49 (86.0%)	8 (14.0%)	0.55^a^
≥6	16	13 (81.3%)	3 (18.8%)	
Ulceration				
Absent	73	60 (82.2%)	13 (17.8%)	0.89^a^
Present	30	25 (83.3%)	5 (16.7%)	
Sentinel lymph node status				
Negative	88	70 (79.5%)	18 (20.5%)	0.07^b^
Positive	15	15 (100%)	0 (0.0%)	
AJCC Stage				
Tis	12	11 (91.7%)	1 (8.3%)	
Stage 1–2	74	57 (77.0%)	17 (23.0%)	0.04^b^*
Stage 3–4	17	17 (100%)	0 (0.0%)	
Lymphovascular invasion				
Absent	93	75 (80.6%)	18 (19.4%)	0.20^b^
Present	10	10 (100%)	0 (0.0%)	
Desmoplastic component				
Absent	94	76 (80.9%)	18 (19.1%)	0.35^b^
Present	9	9 (100%)	0 (0.0%)	
Neurotropism				
Absent	87	69 (79.3%)	18 (20.7%)	0.07^b^
Present	16	16 (100%)	0 (0.0%)	
Regression				
Absent	86	72 (83.7%)	14 (16.3%)	0.49^b^
Present	17	13 (76.5%)	4 (23.5%)	
Infiltrating lymphocyte				
Absent	14	12 (85.7%)	2 (14.3%)	
Non-brisk	80	66 (82.5%)	14 (17.5%)	0.90^b^
Brisk	9	7 (77.8%)	2 (22.2%)	
Predominant cell type^c^				
Epithelioid	40	25 (62.5%)	15 (37.5%)	
Spindle	5	5 (100%)	0 (0.0%)	<0.001^b^*
Mixed	39	38 (97.4%)	1 (2.6%)	

AJCC, American Joint Committee on Cancer; NM, nodular melanoma; SSM, superficial spreading melanoma

^a^Comparison by chi-square test.

^b^Comparison by Fisher’s exact test.

^c^Nineteen cases with unknown predominant cell type excluded for this stratum.

**Fig 1:**
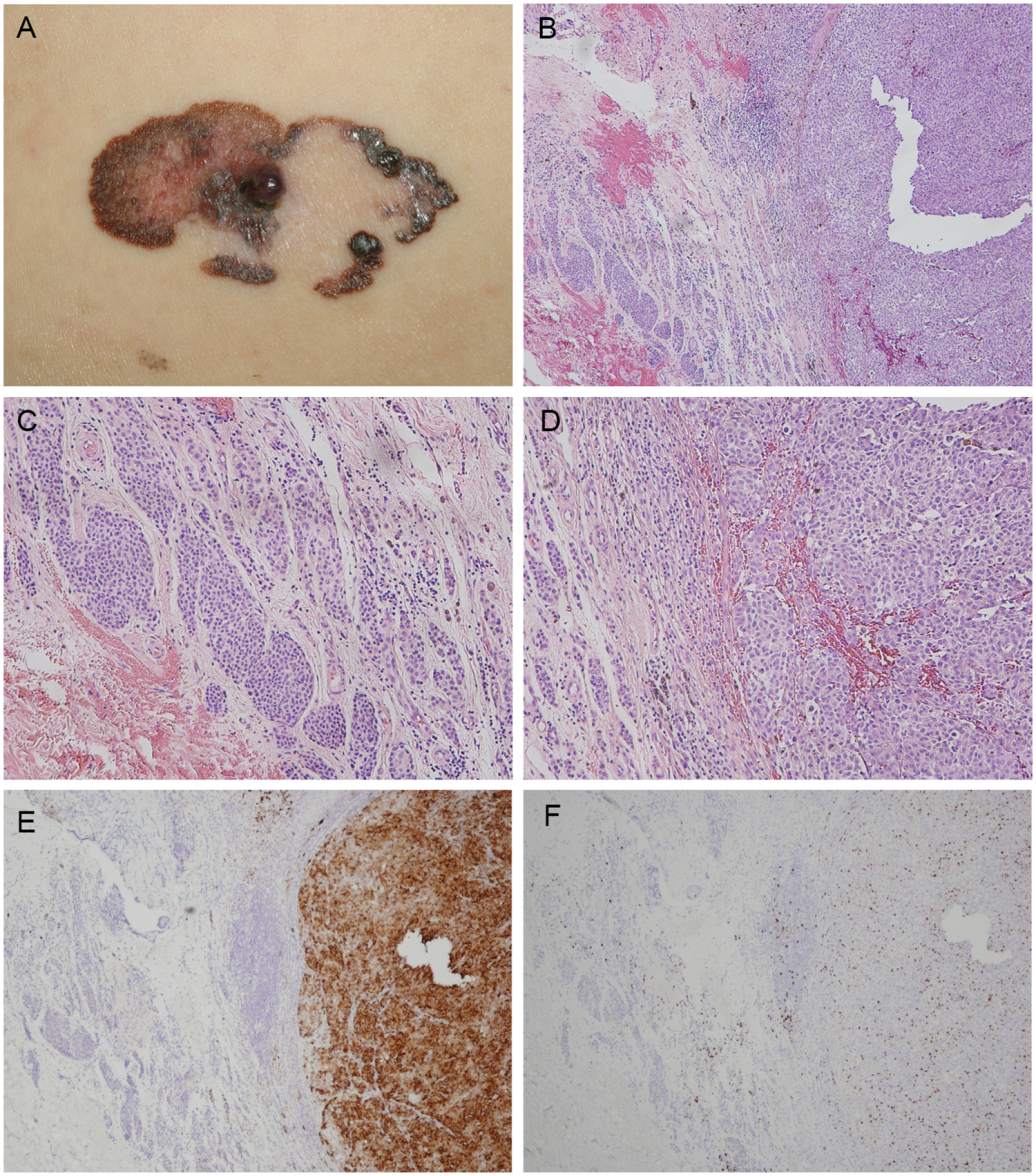
Superficial spreading melanoma arising from a nevus. (A) A 63-year-old man presented with a 5 x 2.5 cm plaque on the left flank with irregular scalloped borders, mottled variegate color, large areas of regression, and a small nodule in the center. (B) Low-powered photomicrograph demonstrating a nodular lesion composed of tumor cells arranged in nests in the dermis with a Breslow thickness of 3 mm. Mitotic figures are numerous. Original magnification x 40. (C) Conventional dermal nevus nests can be noted adjacent to the tumor mass. Original magnification x 100. (D) High-powered photomicrograph reveals nests of tumor cells with vesicular nuclei and prominent nucleoli. Original magnification x 100. (E) Melanoma cells stained positive and nevus cells negative for HMB-45. Original magnification x 40. (F) Melanoma cells stained positive for Ki-67. Original magnification x 40.

With regard to the different types of melanomas, ALMs accounted for 70.9% of the cases, SSMs accounted for 20.4%, NMs accounted for 5.8%, mucosal melanomas accounted for 1.9%, desmoplastic melanomas accounted for 1%, and LMMs accounted for 0%. In this cohort, 17.5% (18/103) of the patients had primary melanomas associated with a nevus pathologically (Table 1). In patients under 65 years of age, 55% (11/20) of the non-ALMs and 4% (1/25) of the ALMs were associated with nevi. This implied that, for patients who did not have ALM and were under 65 years of age, the percentage of nevus-associated melanomas was significantly higher than the patients who had ALMs and were under 65 years of age (*p*<0.001, Fisher’s exact test). In patients older than 65 years of age, there was no significant difference between ALMs and non-ALMs in terms of the percentage of nevus-associated melanomas in each group. 44.4% (8/18) of nevus-associated melanomas were associated with dysplastic nevi, 33.3% (6/18) were associated with junctional/compound/intradermal nevi, and 22.2% (4/18) were associated with congenital nevi. According to the medical records, 72.2% (13/18) of the patients in this nevus-associated melanoma group reported that their melanomas had arisen from a pre-existing nevus. Four of those patients reported that their melanomas had arisen from a congenital nevus, four others reported that the nevus had been present for as long as they could remember, and the remaining patients had a mean lesion duration of 8 years. Of the 21 patients with SSMs, 9 patients (42.9%) were reported to have melanomas with nevi (Fig 1). However, only 9.6% (7/73) of the ALMs were found to have associated melanocytic nevi, while none of the mucosal or desmoplastic melanomas were associated with a nevus.

Assessment of clinical and pathological features found a significant correlation of nevus-associated melanomas with intermittent solar exposure, younger patient age, locations on the trunk and extremities, the SSM subtype, and early stage (Table 1). The nevus-associated melanomas exhibited a trend toward thinner tumors and negative sentinel lymph node status for nevus-associated melanomas (*p* = 0.06 and 0.07, Table 1), and when the Breslow thickness values were stratified into two groups (≤1.0 mm and >1.0 mm), the de novo melanomas were significantly associated with tumor thicknesses equal to or greater than 1.0 mm (*p* = 0.03, Fisher’s exact test).

According to the histologic variables, the epithelioid cell type was significantly correlated with nevus-associated melanomas (*p* <0.001, Table 1). However, when the two groups were compared for ulceration, mitotic count, lymphovascular invasion, desmoplasia, neurotropism, tumor regression, and lymphocytic infiltrate, no statistically significant difference was found.

**Fig 2:**
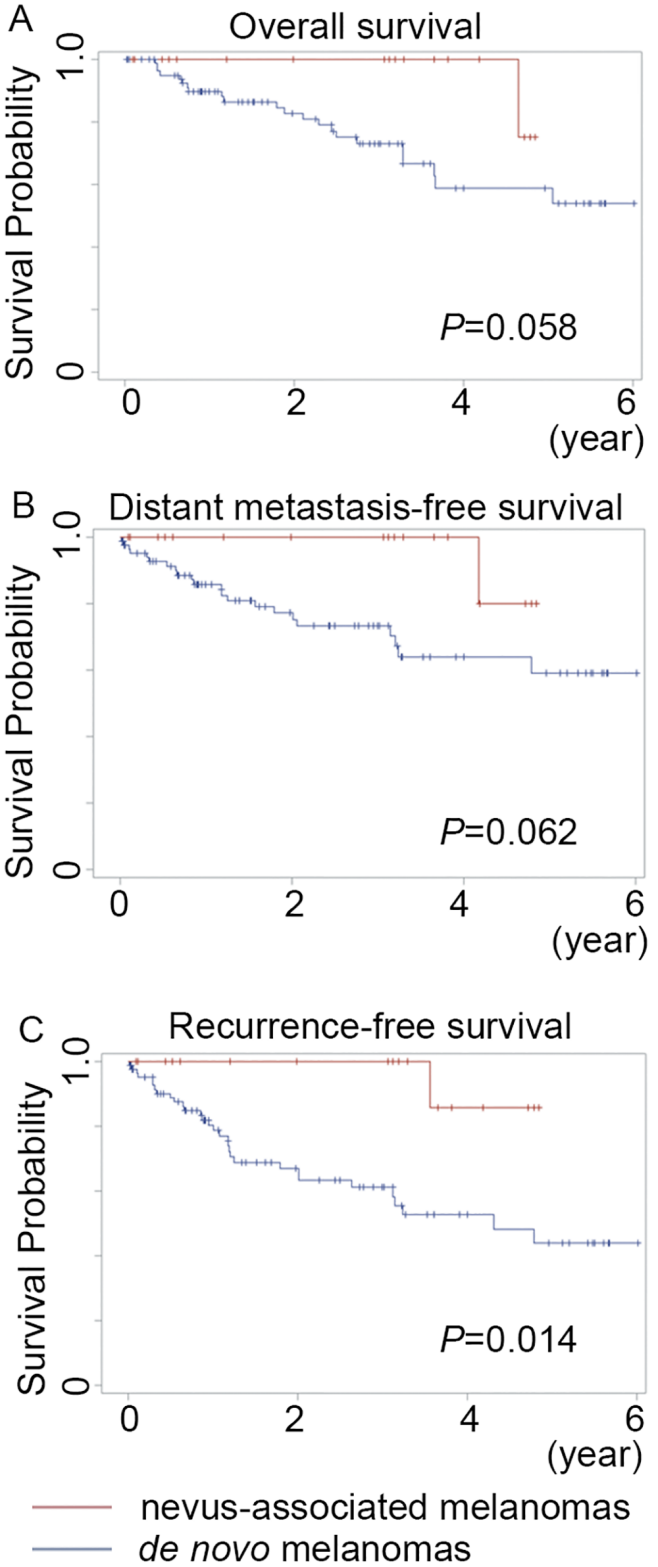
Kaplan-Meier curves of survival for 103 primary melanoma patients. No overall (A) and distant metastasis-free survival (B) differences were found between patients with de novo and nevus-associated melanomas (*p* = 0.058 and 0.062, respectively). (C) Patients with melanomas arising in association with a pre-existing nevus had better recurrence-free survival than those with de novo melanomas (*p* = 0.014).

The 5-year OS rate for all 103 of the melanoma patients was 63.7%. When stratified by de novo (n = 85) and nevus-associated (n = 18) melanomas, the 5-year OS rates of the patients were 60.4% and 75%, respectively. Kaplan-Meier curves revealed no significant differences in OS or DMFS between melanomas associated with a nevus and melanomas occurring de novo (*p* = 0.058 and 0.062, respectively, log rank test) (Fig 2A & 2B). In contrast, patients with nevus-associated melanomas had significantly better RFS than those with de novo melanomas (Fig 2C; *p* = 0.014, log rank test). Lymph node status (HR, 5.47; 95% CI, 2.37–12.65; *p*<0.001), ulceration (HR, 4.67; 95% CI, 2.04–10.72; *p*<0.001), and mitotic count (HR, 1.06; 95% CI, 1.01–1.11; *p* = 0.03) were associated with OS in a univariate analysis (Table 2). Univariate analysis further revealed that metastatic nodes (HR, 9.12; 95% CI, 3.97–20.93; *p*<0.001), ulceration (HR, 4.19; 95% CI, 1.81–9.74; *p* = 0.001), mitotic count (HR, 1.07; 95% CI, 1.02–1.12; *p* = 0.009), and subtype (others vs. NM) (HR, 0.18; 95% CI, 0.05–0.64; *p* = 0.008) predicted an unfavorable DMFS (S2 Table). Univariate analysis results also showed that certain factors were significantly associated with RFS, including the presence of an associated nevus (HR, 0.12; 95% CI, 0.02–0.91; *p* = 0.04), lymph node status (HR, 7.06; 95% CI, 3.4–14.66; *p*<0.001), ulceration (HR, 3.37; 95% CI, 1.65–6.86; *p*<0.001), mitotic count (HR, 1.06; 95% CI, 1.01–1.11; *p* = 0.009) and melanoma subtype (others vs. NM) (HR, 0.18; 95% CI, 0.05–0.62; *p* = 0.007); there was no significant association, however, of RFS with age, sex, location, or solar exposure (S3 Table). According to the multivariate analysis, meanwhile, melanoma subtype was an independent prognostic variable for DMFS and RFS, while lymph node status and ulceration remained the most crucial prognostic factors for predicting OS, DMFS, and RFS.

**Table 2:** Univariate and multivariate analysis of risk factors associated with overall survival.

Variables	Univariate HR (95% CI)	Univariate *P*-value	Multivariate HR (95% CI)	Multivariate *P*-value
Age, y	1.00 (0.97–1.02)^a^	0.81		
Sex (men vs. women)	1.10 (0.49–2.46)	0.81		
Location (others vs. extremity)	2.59 (0.35–19.21)	0.35		
Solar exposure (intermittent/chronic vs. no)	1.00 (0.37–2.7)	0.99		
Lymph node status (present vs. absent)	5.47 (2.37–12.65)	<0.001*	3.85 (1.49–9.93)	0.005*
Ulcer (present vs. absent)	4.67 (2.04–10.72)	<0.001*	4.12 (1.67–10.16)	0.002*
Thickness, mm	1.02 (0.97–1.08)^a^	0.42		
Mitosis/mm^2^	1.06 (1.01–1.11)^a^	0.03*	1.00 (0.93–1.07)^a^	0.99
Subtype (others vs. NM)	0.27 (0.06–1.18)	0.08		
Associated nevus (present vs. absent)	0.18 (0.02–1.34)	0.09	0.25 (0.03–1.95)	0.19

Abbreviations: CI, confidence intervals; HR, hazard ratio; NM, nodular melanoma.

^a^Hazard ratio corresponds to a 1-year increase in age, 1-mm increase in thickness and 1- mitosis increase per mm^2^

**Fig 3:**
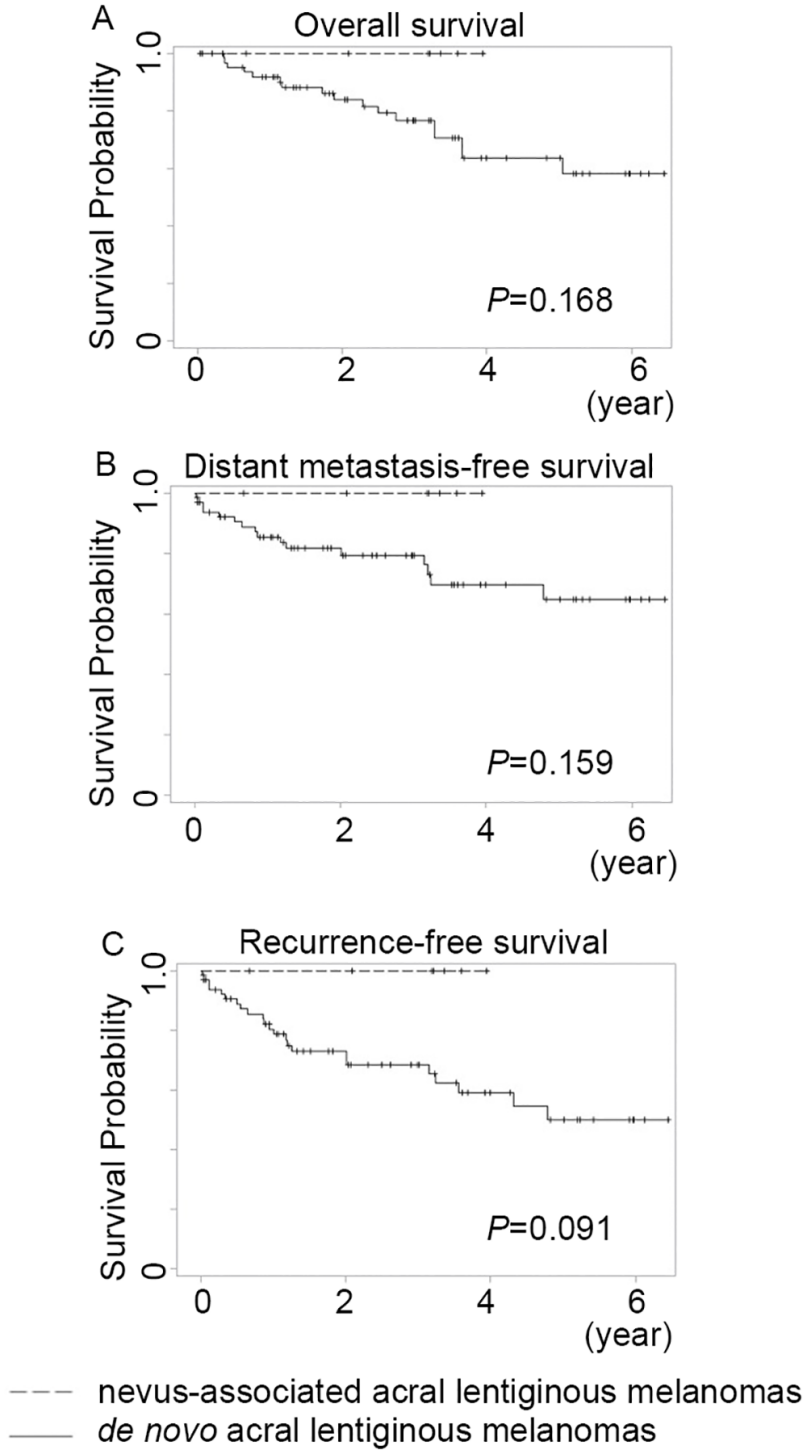
Kaplan-Meier curves of survival for 73 patients with primary acral lentiginous melanomas. No overall (A), distant metastasis-free survival (B), and recurrence-free survival (C) differences were found between patients with de novo and nevus-associated acral lentiginous melanomas (*p* = 0.168, 0.159, and 0.091, respectively, log rank test).

The data for the 73 ALMs are summarized in Table 3. The mean and median tumor thicknesses of the ALMs were 3.4 and 2.1 mm (range 0.25–19 mm). Of the seven nevus-associated ALMs, 57.1% (4/7) were associated with dysplastic nevi and 42.9% (3/7) were associated with acral type junctional/compound nevi. None was associated with a congenital nevus. In terms of clinical and histological characteristics, the nevus-associated ALMs did not differ significantly from those arising de novo. The 5-year OS rate for the 73 patients with ALMs was 66.5%, with the five-year OS rates for nevus-associated ALM patients (n = 7) and de novo ALMs patients (n = 66) being 100% and 63.6%, respectively. Kaplan-Meier curves revealed no significant OS, DMFS, and RFS differences between patients with nevus-associated ALMs and patients with de novo ALMs (*p* = 0.168, 0.159, and 0.091, respectively, log rank test) (Fig 3).

**Table 3:** Clinical and histologic characteristics in acral lentigious melanomas.

Characteristic	Total	De novo	Nevus-associated	*P*-value^a^
Age (years)				
<65	25	24 (96.0%)	1 (4.0%)	0.41
≥65	48	42 (87.5%)	6 (12.5%)	
Sex				
Female	34	30 (88.2%)	4 (11.8%)	0.70
Male	39	36 (92.3%)	3 (7.7%)	
Thickness of invasive melanomas (n = 63), mm				0.43
≤ 1.0	11	9 (81.8%)	2 (18.2%)	
1.01–2.0	19	17 (89.5%)	2 (10.5%)	
2.01–4.0	18	16 (88.9%)	2 (11.1%)	
>4.0	15	15 (100%)	0 (0.0%)	
Mitosis/mm^2^				
<1	19	17 (89.5%)	2 (10.5%)	
1–5	43	40 (93.0%)	3 (7.0%)	0.40
≥6	11	9 (81.8%)	2 (18.2%)	
Ulceration				
Absent	50	46 (92.0%)	4 (8.0%)	0.67
Present	23	20 (87.0%)	3 (13.0%)	
Sentinel lymph node status				
Negative	61	54 (88.5%)	7 (11.5%)	0.59
Positive	12	12 (100%)	0 (0.0%)	
AJCC Stage				
Tis	10	9 (90.0%)	1 (10.0%)	
Stage 1–2	49	43 (87.8%)	6 (12.2%)	0.49
Stage 3–4	14	14 (100%)	0 (0.0%)	
Lymphovascular invasion				
Absent	66	59 (89.4%)	7 (10.6%)	1.00
Present	7	7 (100%)	0 (0.0%)	
Desmoplastic component				
Absent	66	59 (89.4%)	7 (10.6%)	1.00
Present	7	7 (100%)	0 (0.0%)	
Neurotropism				
Absent	60	53 (88.3%)	7 (11.7%)	0.34
Present	13	13 (100%)	0 (0.0%)	
Regression				
Absent	64	57 (89.1%)	7 (10.9%)	0.59
Present	9	9 (100%)	0 (0.0%)	
Infiltrating lymphocyte				
Absent	12	12 (100%)	0 (0%)	
Non-brisk	57	50 (87.7%)	7 (12.3%)	0.56
Brisk	4	4 (100%)	0 (0%)	
Predominant cell type^b^				
Epithelioid	23	18 (78.3%)	5 (21.7%)	
Spindle	5	5 (100%)	0 (0%)	0.09
Mixed	32	31 (96.9%)	1 (3.1%)	

AJCC, American Joint Committee on Cancer; NM, nodular melanoma; SSM, superficial spreading melanoma

^a^ Comparison by Fisher’s exact test.

^b^ Thirteen cases with unknown predominant cell type excluded for this stratum.

## Discussion

Melanomas have usually been described in terms of their association with melanocytic nevi [2, 6, 13]. One meta-analysis study for a given time period showed that 32% of melanomas presented in association with a nevus histopathologically [6]. In the current report, only 17.5% of the patients were found to have melanomas with nevi, a rate which was much lower than that reported by a previous study of Caucasian patients [6]. This can be explained by the fact that over 70% of the melanomas of the patients included in this study were ALMs, which are seldom associated with a nevus [2]. The risk factors of ALMs include mechanical stress, penetrative injury, heavy exposure to agricultural chemicals, and ethnicity [14–16]. The mean Breslow thickness of all the melanomas in this investigation was 3.7 mm, which was much thicker than the thicknesses reported in previous reviews of Caucasian patients, suggesting that melanomas tend to have greater thickness in Asian patients [17, 18]. Although over half of all melanomas in Asians are ALMs, ALMs account for only 3–10% of all melanoma patients among Caucasians [19–22]. With regard to the incidence of nevus-associated melanomas in each subtype, the results of this study were consistent with previous findings [6, 12, 23]. It has been proposed that histologic subtype seems to be the primary factor in whether a melanoma is nevus-associated or de novo, with SSMs more likely to emerge in a nevus than other types of melanomas [6, 12]. In patients under 65 years of age, non-ALMs were significantly associated with a higher percentage of nevus-associated melanomas.

Consistent with previous reports, the present study found that patients with nevus-associated melanomas tended to be younger than those with de novo melanomas [6, 18, 24]. The early age at onset of nevus-associated melanomas suggests that pigmentation-related genes are important factors in malignant melanoma susceptibility [25]. This age issue may also be due to the smaller numbers of moles in older individuals, which may in turn be due to a time-related involution of nevi and the presence of fewer nevus precursors from which melanomas may evolve [26]. The finding of this study that nevus-associated melanomas developed preferentially on areas subjected to intermittent sun exposure (i.e., the trunk and extremities) is in line with earlier reports [1, 12]. This study also found that nevus-associated melanomas were more likely to be associated with thinner tumors, and thus had a better prognosis in general. Although some previous studies have reported that Breslow thickness did not differ significantly in nevus-associated versus de novo melanomas, thinner tumors have been found to be associated with an increased likelihood of nevus-associated melanoma in other reports [6, 11, 12, 24, 26–29]. More specifically, nevus-associated melanomas were reported to be more frequent in thinner tumors, possibly owing to the obliteration of nevocytic remnants in more advanced melanomas [12, 24]. Nevus-associated melanomas have also been reported to be significantly associated with epithelioid cells morphologically, which could be related to the fact that half of nevus-associated melanomas are SSMs [12, 23]. Histologically, the de novo melanomas tend to present with neurotropism, and neurotropism is most likely to be associated with poor survival in desmoplastic melanomas and other melanoma types [30]. Neurotropism is also linked with an increase in the frequency of local recurrence of melanomas [30]. This could partly explain the result of better RFS in nevus-associated melanomas. In any case, these findings suggest there are differences in the biology of nevus-associated and de novo melanomas.

The prognostic significance of nevus-associated melanomas has continued to be controversial in past reports [12]. Friedman *et al*. reported that patients with nevus-associated melanomas had decreased rates of metastasis and death, but only for melanomas between 0.75 and 1.69 mm and greater than 3.6 mm in thickness [7]. Kaddu *et al*. suggested that patients with nevus-associated melanomas had a favorable clinical outcome, although there was no significant difference in survival between the two groups when stratified by tumor thickness [8]. Meanwhile, Rhodes *et al*., using a univariate analysis, reported that cases of nevus-associated melanomas were less likely to result in metastasis and death [27]. However, the aforementioned studies did not consider certain factors that are now known to influence survival [5]. More recently, a study by Lin *et al*. found that there was no OS difference between nevus-associated and de novo melanomas [6]. In contrast, Gymerman *et al*. found that patients with de novo melanomas had shorter OS than patients whose melanomas arose in association with a nevus, but they did not include an analysis of sentinel node biopsy results [18]. Gymerman *et al*. also reported de novo melanomas were associated with stages greater than stage I [18]. Relatedly, the current study found that nevus-associated melanomas were associated with early stages and tended to have less positive sentinel lymph nodes. We also combined OS, DMFS, and RFS analysis in this study and found that the nevus-associated melanomas were significantly associated with better RFS, with association with a nevus being significantly correlated with better RFS in the univariate analysis. Due to the relatively short follow-up period, however, nevus-associated melanomas only had a trend toward better OS and DMFS, and unsurprisingly, the multivariate analysis did not find nevus-associated melanoma to be an independent prognostic factor of OS, DMFS, or RFS. The lower survival rates among our patients compared with Caucasian patients were likely in part due to their advanced stages of disease at diagnosis, the lack of awareness of this disease, and further delaying of potentially curative surgical treatment [13, 31].

There have been few opportunities to investigate patients with ALMs associated with nevi, and the precise frequency of nevus-associated ALMs remains unknown [2]. Therefore, we sought to present our cases of nevus-associated ALMs in this study. In the current study, nevus-associated melanomas accounted for 9.6% of all ALMs, which was comparable the rates reported in other studies [12, 18]. None of the nevus-associated ALMs had positive nodes, advanced stages, lymphovascular invasion, desmoplastic component, neurotropism, or regression. However, there was no statistically significant difference noted between the nevus-associated and de novo ALMs with regard to clinical and pathological variables. Although the patients with nevus-associated ALMs had a better 5-year OS rate compared to those with de novo ALMs, there was no significant difference between nevus-associated and de novo ALMs when using the log-rank test. This lack of difference in the characteristics and survival in the ALM group may be due to the fact that the number of patients with nevus-associated lesions in this group was too small to exhibit any difference.

The limitations of this study include its retrospective design and single-center site. Cutaneous melanoma is a disease that occurs only rarely in Asians, with an average annual age-adjusted incidence 1 in 100,000 [31]. Therefore, we could only include a limited number of cases in the analysis, and although this study, among those published to date, has one of the largest samples for a prognostic investigation in Asia, a larger series may be able to better determine the prognostic significance of nevus-associated melanomas in Asian patients. Meanwhile, whether the data presented in this study are applicable to nevus-associated melanomas in other populations needs to be confirmed through further reviews including other Asians, Caucasians, and Africans. Another limitation of this study is that the nevus-associated melanomas investigated were defined on a pathological basis. In fact, however, the criteria for defining melanomas as nevus-associated melanomas are a subject of some controversy [32]. Some reports have diagnosed nevus-associated melanomas based on patient anamnesis, whereas others have regarded the pathologically proven presence of nevus cells as diagnostic for nevus-associated melanomas [7, 28, 32]. In this regard, it should be noted that melanoma cells may change their shapes into nevus-like cells, such that a pathological examination alone may not be enough to judge for certain whether a given melanoma is associated with a nevus [7, 32]. Furthermore, while a patient history can be used to determine if a melanoma has arisen from a nevus, it can also lead to misdiagnosis due to the questionable accuracy of patient recall of a preexisting lesion [32]. Moreover, melanoma cells can destroy nevus cells as they grow, and it is not always possible to clinically observe the nevus in advanced cases of melanoma. Further studies are thus necessary in order to draw firm conclusions regarding the difference between nevus-associated and de novo melanomas diagnosed clinically and pathologically.

In summary, nevus-associated melanomas were found to be uncommon among the ALMs patients investigated in this study. There was no statistically significant difference between the nevus-associated and de novo ALMs with regard to clinicopathological factors and survival. Finally, because over half of all melanomas in Asians are ALMs, Asians are less likely than Caucasians to have nevus-associated melanomas.

## Supporting information

S1 Table Data from a total of 103 cases with primary melanoma. (XLSX)

S2 Table Univariate and multivariate analysis of risk factors associated with distant metastasis-free survival. (DOCX)

S3 Table Univariate and multivariate analysis of risk factors associated with recurrence-free survival. (DOCX)
