# Supplementary material for: Clinicopathological features and prognosis of patients with de novo versus nevus-associated melanoma in Taiwan
Source: PLoS One. 2017 May 4;12(5):e0177126. doi: 10.1371/journal.pone.0177126 (PMC5417671; doi:10.1371/journal.pone.0177126)
Supplement: S2 Table — (DOCX) [file pone.0177126.s002.docx]

**S2 Table. Univariate and multivariate analysis of risk factors associated with distant metastasis-free survival**

| **Variables** | **Univariate HR**  **(95% CI)** | **Univariate *P*-value** | **Multivariate HR**  **(95% CI)** | **Multivariate *P*-value** |
| --- | --- | --- | --- | --- |
| Age, y | 1.00 (0.97-1.03)^a^ | 0.97 |  |  |
| Sex (men vs women) | 1.21 (0.53-2.76) | 0.65 |  |  |
| Location (others vs. extremity) | 2.72 (0.37-20.17) | 0.33 |  |  |
| Solar exposure (intermittent/chronic vs. no) | 1.00 (0.37-2.71) | 1.00 |  |  |
| Lymph node status (present vs. absent) | 9.12 (3.97-20.93) | <0.001^*^ | 6.59 (2.54-17.12) | <0.001^*^ |
| Ulcer (present vs. absent) | 4.19 (1.81-9.74) | 0.001^*^ | 5.00 (1.80-13.93) | 0.002^*^ |
| Thickness, mm | 1.02 (0.98-1.08)^a^ | 0.34 |  |  |
| Mitosis/mm^2^ | 1.07 (1.02-1.12)^a^ | 0.009^*^ | 0.97 (0.90-1.04)^a^ | 0.38 |
| Subtype (others vs. NM) | 0.18 (0.05-0.64) | 0.008^*^ | 0.19 (0.04-0.82) | 0.03^*^ |
| Associated nevus (present vs. absent) | 0.18 (0.03-1.36) | 0.10 | 0.32 (0.04-2.47) | 0.27 |

Abbreviations: CI, confidence intervals; HR, hazard ratio; NM, nodular melanoma.

^a^Hazard ratio corresponds to a 1-year increase in age, 1-mm increase in thickness and 1- mitosis increase per mm^2^
