# Supplementary material for: Clinicopathological features and prognosis of patients with de novo versus nevus-associated melanoma in Taiwan
Source: PLoS One. 2017 May 4;12(5):e0177126. doi: 10.1371/journal.pone.0177126 (PMC5417671; doi:10.1371/journal.pone.0177126)
Supplement: S3 Table — (DOCX) [file pone.0177126.s003.docx]

**S3 Table. Univariate and multivariate analysis of risk factors associated with recurrence-free survival**

| **Variables** | **Univariate HR**  **(95% CI)** | **Univariate *P*-value** | **Multivariate HR (95% CI)** | **Multivariate *P*-value** |
| --- | --- | --- | --- | --- |
| Age, y | 1.01 (0.98-1.03)^a^ | 0.67 |  |  |
| Sex (men vs. women) | 1.21 (0.6-2.43) | 0.60 |  |  |
| Location (others vs. extremity) | 4.02 (0.55-29.49) | 0.17 |  |  |
| Solar exposure (intermittent/chronic vs. no) | 0.83 (0.34-2.02) | 0.68 |  |  |
| Lymph node status (present vs. absent) | 7.06 (3.40-14.66) | <0.001^*^ | 5.49 (2.36-12.74) | <0.001^*^ |
| Ulcer (present vs. absent) | 3.37 (1.65-6.86) | <0.001^*^ | 3.52 (1.54-8.04) | 0.003^*^ |
| Thickness, mm | 1.04 (1.00-1.07)^a^ | 0.054 |  |  |
| Mitosis/mm^2^ | 1.06 (1.01-1.11)^a^ | 0.009^*^ | 0.97 (0.91-1.03)^a^ | 0.28 |
| Subtype (others vs. NM) | 0.18 (0.05-0.62) | 0.007^*^ | 0.12 (0.03-0.55) | 0.006^*^ |
| Associated nevus (present vs. absent) | 0.12 (0.02-0.91) | 0.04^*^ | 0.18 (0.02-1.32) | 0.09 |

Abbreviations: CI, confidence intervals; HR, hazard ratio; NM, nodular melanoma.

^a^Hazard ratio corresponds to a 1-year increase in age, 1-mm increase in thickness and 1- mitosis increase per mm^2^
